# Supplementary material for: Interplay between the human gut microbiome and host metabolism
Source: Nat Commun. 2019 Oct 3;10:4505. doi: 10.1038/s41467-019-12476-z (PMC6776654; doi:10.1038/s41467-019-12476-z)
Supplement: Supplementary file 1 — Supplementary Information [file 41467_2019_12476_MOESM1_ESM.pdf]

Interplay between the human gut microbiome and host metabolism

Visconti *et al.*

Supplementary Methods

Faecal Metabolomic Profiling

Detailed description of the faecal metabolic profiling is reported here as it appears in the original manuscripts[1].

Sample preparation for global metabolomics

Samples were stored at –80 °C until processing. Sample preparation was carried out as described previously at Metabolon, Inc[2]. Lyophilized fecal samples were extracted at a constant per-mass basis. Briefly, recovery standards were added before the first step in the extraction process for quality-control purposes. To remove protein, dissociate small molecules bound to protein or trapped in the precipitated protein matrix, and recover chemically diverse metabolites, proteins were precipitated with methanol under vigorous shaking for 2 min (Glen Mills Genogrinder 2000), then centrifuged. The resulting extract was divided into five fractions: (i) acidic positive-ion conditions chromatographically optimized for more hydrophilic compounds; (ii) acidic positive-ion conditions chromatographically optimized for more hydrophobic compounds; (iii) basic negative-ion-optimized conditions with a separate dedicated C18 column; (iv) negative ionization after elution from a HILIC column; (v) reserved for backup.

Three types of controls were analyzed in concert with the experimental samples: a pooled sample generated from a small portion of each experimental sample of interest served as a technical replicate throughout the platform run; extracted water samples served as process blanks; and a cocktail of standards spiked into every analyzed sample allowed for instrument performance monitoring. Instrument variability was determined by calculation of the median relative s.d. (RSD) for the standards that were added to each sample before injection into the mass spectrometers (median RSDs were determined to be 5%; n = 31 standards). Overall process variability was determined by calculating the median RSD for all endogenous metabolites (i.e., noninstrument standards) present in 90% or more of the pooled technical-replicate samples (median RSD = 12%, n = 832 metabolites). Experimental samples and controls were randomized across the platform run.

Mass spectrometry analysis

Extracts were subjected to UPLC–MS/MS[3]. The chromatography was standardized, and no further changes were made after the method was validated. As part of Metabolon's general practice, all columns were purchased from a single manufacturer's lot at the outset of experiments. All solvents were similarly purchased in bulk from a single manufacturer's lot in sufficient quantity to complete all related experiments. For each sample, vacuum-dried samples were dissolved in injection solvent containing eight or more injection standards at fixed concentrations, depending on the platform. The internal standards were used to ensure both injection and chromatographic consistency. Instruments were tuned and calibrated for mass resolution and mass accuracy daily. All methods used a Waters Acquity UPLC and a Thermo Scientific Q-Exactive high-resolution/accurate mass spectrometer interfaced with a heated electrospray ionization (HESI-II) source and an Orbitrap mass analyzer operated at 35,000 mass resolution. The sample extract was dried, then reconstituted in solvents compatible with each of the four methods. Each reconstitution solvent contained a series of standards at fixed concentrations to ensure injection and chromatographic consistency. One aliquot was analyzed by using acidic positive-ion conditions, which were chromatographically optimized for relatively hydrophilic compounds. In this method, the

extract was gradient eluted from a C18 column (Waters UPLC BEH C18, 2.1 × 100 mm, 1.7 µm) with water and methanol containing 0.05% perfluoropentanoic acid and 0.1% formic acid. Another aliquot was also analyzed by using acidic positive-ion conditions; however, it was chromatographically optimized for relatively hydrophobic compounds. In this method, the extract was gradient eluted from the same aforementioned C18 column with methanol, acetonitrile, water, 0.05% perfluoropentanoic acid, and 0.01% formic acid, and was operated at an overall higher organic content. Another aliquot was analyzed by using basic negative-ion-optimized conditions and a separate dedicated C18 column. The basic extracts were gradient eluted from the column with methanol and water, as well as 6.5 mM ammonium bicarbonate at pH 8. The fourth aliquot was analyzed via negative ionization after elution from a HILIC column (Waters UPLC BEH Amide 2.1 × 150 mm, 1.7 µm) with a gradient consisting of water and acetonitrile with 10 mM ammonium formate, pH 10.8. The MS analysis alternated between MS and data-dependent MSn scans using dynamic exclusion. The scan range varied slightly between methods but covered 80–1,000 m/z.

Compound identification, quantification, and data curation

Metabolites were identified by automated comparison of the ion features in the experimental samples to a reference library of chemical standard entries that included retention time, molecular weight (m/z), preferred adducts, and in-source fragments as well as associated MS spectra, and were curated by visual inspection for quality control in software developed at Metabolon[3,4]. Identification of known chemical entities was based on comparison to metabolomic library entries of purified standards. Commercially available purified standard compounds have been acquired and registered into LIMS for distribution to the various UPLC-MS/MS platforms for determination of their detectable characteristics. Additional mass-spectral entries have been created for structurally unnamed biochemicals, which have been identified on the basis of their recurrent nature (both chromatographic and mass spectral). These compounds have the potential to be identified by future acquisition of a matching purified standard or by classical structural analysis. Peaks were quantified through area-under-the-curve analysis. Raw area counts for each metabolite in each sample were normalized to correct for variation resulting from instrument interday tuning differences by the median value for each run day, and the medians were therefore set to 1.0 for each run. This procedure preserved variation among samples but allowed metabolites of widely different raw peak areas to be compared on a similar graphical scale.

A total of 1,116 different metabolites were measured in the 786 fecal samples, of which 210 metabolites were observed in less than 20% of the samples and thus were excluded from further analysis because of lack of power. 345 metabolites were observed in more than 20% but less than 80% of the samples and were thus analyzed qualitatively as dichotomous traits (observed in a sample versus not observed). The remaining 570 metabolites, which were observed in at least 80% of all samples, were scaled by run-day medians, log-transformed and scaled to uniform mean 0 and s.d. 1 and analyzed quantitatively. Metabolite ratios were calculated from the run-day median-normalized metabolite levels and subsequently log-transformed and scaled to a mean of 0 and s.d. of 1.

We analyzed effects of sample storage time (i) in the refrigerator before samples were frozen and (ii) in the freezer before further analysis. To this end, we regressed metabolite concentrations against storage times. After correcting for multiple testing, we found significant storage effects on seven metabolites (FDR <0.05). We thus corrected all further analyses for both storage time in the refrigerator and freezer, to avoid spurious results. Despite correcting

all models for the storage time, we cannot ultimately eliminate a potential confounding effect due to storage time, and future studies should investigate the influence of storage time on fecal metabolites.

Blood Metabolomic Profiling

Detailed description of the blood metabolic profiling is reported here as it appears in the original manuscripts[5].

Metabolite profiling

The non-targeted metabolomics analysis was performed at Metabolon (Durham, North Carolina, USA) on a platform consisting of four independent ultra-high-performance liquid chromatography–tandem mass spectrometry (UPLC–MS/MS) instruments. Samples were prepared using the automated MicroLab STAR system from Hamilton Company. Several recovery standards were added before the first step in the extraction process for quality control purposes. To remove protein, to dissociate small molecules bound to protein or trapped in the precipitated protein matrix, and to recover chemically diverse metabolites, proteins were precipitated with methanol under vigorous shaking for 2 min (Glen Mills GenoGrinder 2000) followed by centrifugation. The resulting extract was divided into five fractions: two for analysis by two separate reverse-phase (RP)/UPLC–MS/MS methods with positive-ion-mode electrospray ionization (ESI), one for analysis by RP/UPLC–MS/MS with negative-ion-mode ESI, one for analysis by HILIC/UPLC–MS/MS with negative-ion-mode ESI, and one reserved for backup. Samples were placed briefly on a TurboVap (Zymark) to remove the organic solvent. The sample extracts were stored overnight under nitrogen before preparation for analysis. Several types of controls were analyzed in concert with the experimental samples: a pooled matrix sample generated by taking a small volume of each experimental sample (or, alternatively, a pool of well-characterized human plasma) served as a technical replicate throughout the data set; extracted water samples served as process blanks; and a cocktail of quality control standards that were carefully chosen not to interfere with the measurement of endogenous compounds was spiked into every analyzed sample, allowed instrument performance monitoring and aided chromatographic alignment. Instrument variability was determined by calculating the median relative standard deviation (RSD) for the standards that were added to each sample before injection into the mass spectrometers. Overall process variability was determined by calculating the median RSD for all endogenous metabolites (i.e., non-instrument standards) present in 100% of the pooled-matrix samples. Experimental samples were randomized across the platform run with quality control samples spaced evenly among the injections. All methods used a Waters ACQUITY ultra-performance liquid chromatographer and a Thermo Scientific Q-Exactive high-resolution mass spectrometer interfaced with a heated electrospray ionization (HESI-II) source and Orbitrap mass analyzer operated at 35,000 mass resolution. The sample extract was dried and then reconstituted in solvents compatible with each of the four methods. Each reconstitution solvent contained a series of standards at fixed concentrations to ensure injection and chromatographic consistency. One aliquot was analyzed using acidic positive-ion conditions, chromatographically optimized for more hydrophilic compounds. In this method, the extract was gradient eluted from a C18 column (Waters UPLC BEH C18–2.1 × 100 mm, 1.7 µm) using water and methanol, containing 0.05% perfluoropentanoic acid (PFPA) and 0.1% formic acid (FA). Another aliquot was also analyzed using acidic positive-ion conditions; however, it was chromatographically optimized for more hydrophobic compounds. In this method, the extract was gradient eluted from the same afore-mentioned C18 column using methanol, acetonitrile, water, 0.05% PFPA and 0.01% FA and was operated at an overall higher

organic content. Another aliquot was analyzed using basic negative-ion-optimized conditions on a separate dedicated C18 column. The basic extracts were gradient eluted from the column using methanol and water, however, with 6.5 mM ammonium bicarbonate at pH 8. The fourth aliquot was analyzed via negative ionization following elution from a HILIC column (Waters UPLC BEH Amide 2.1 × 150 mm, 1.7 µm) using a gradient consisting of water and acetonitrile with 10 mM ammonium formate, pH 10.8. The MS analysis alternated between MS and data-dependent MSn scans using dynamic exclusion. The scan range varied slightly between methods but covered 70–1,000 m/z. Raw data files are archived and extracted as described below. Raw data were extracted, peak identified and quality control processed using Metabolon's hardware and software. These systems are built on a web service platform using Microsoft's .NET technologies, which run on high-performance application servers and fiber-channel storage arrays in clusters to provide active failover and load balancing. Compounds were identified by comparison to library entries of purified standards or recurrent unknown entities. Metabolon maintains a library based on authenticated standards that contains the retention time/index (RI), mass-to-charge ratio (m/z) and chromatographic data (including MS/MS spectral data) on all molecules present in the library. Furthermore, biochemical identifications are based on three criteria: retention index within a narrow RI window of the proposed identification, accurate mass match to the library ±10 ppm, and MS/MS forward and reverse scores between the experimental data and authentic standards. MS/MS scores are based on a comparison of the ions present in the experimental spectrum to the ions present in the library spectrum. While there may be similarities between these molecules based on one of these factors, the use of all three data points can distinguish and differentiate biochemicals. More than 3,300 commercially available purified standard compounds have been acquired and registered into LIMS for analysis on all platforms for determination of their analytical characteristics. Additional mass spectral entries have been created for structurally unnamed biochemicals, which have been identified by virtue of their recurrent nature (both chromatographic and mass spectral). These compounds have the potential to be identified by future acquisition of a matching purified standard or by classical structural analysis. A variety of curation procedures were carried out to ensure that a high-quality data set was made available for statistical analysis and data interpretation. The quality control and curation processes were designed to ensure accurate and consistent identification of true chemical entities and to remove those representing system artifacts, misassignments and background noise. Metabolon data analysts used proprietary visualization and interpretation software to confirm the consistency of peak identification among the various samples. Library matches for each compound were checked for each sample and corrected if necessary.

Co-eluting metabolites

About 60% of metabolites reported by Metabolon are identified and measured across more than one platform (or on the same platform using different quantions), although data from only one of the platforms per metabolite were presented in this manuscript. It is unlikely that a given metabolite would encounter the same influence from co-eluting metabolites, such as ion suppression, on more than one platform. Furthermore, as part of Metabolon's standard QC process, correlations of cross-platform (or cross-library entry) measurements were examined to identify influencing factors. When a poor correlation was identified, data from the suspect platform was not reported back; rather, metabolite data were reported from another platform when at least two other correlated platforms were present.

In some cases, co-eluting metabolites that have identical masses and similar structures are known or suspected to be present in the data set (*e.g.,* 2-hydroxybutyrate and 2-hydroxyisobutyrate or 3-methylglutarate and 2-methylglutarate). These isobaric metabolites are presented in the data tables as "metabolite 1/metabolite 2". These metabolites are not resolvable by the chromatography used for the reported platform and were not measured as resolvable metabolites on the remaining three platforms. The metabolite levels presented, therefore, may represent contributions from one or both of the reported metabolites. Based on findings from many thousands of studies that have been run across the Metabolon platform, these isobaric metabolite entries have been determined to represent a very minor percentage of metabolites reported.

Supplementary references

1. Zierer, J. *et al.* The fecal metabolome as a functional readout of the gut microbiome. *Nat. Genet.* **50**, 790–795 (2018).
2. Bridgewater BR, E. A. High Resolution Mass Spectrometry Improves Data Quantity and Quality as Compared to Unit Mass Resolution Mass Spectrometry in High-Throughput Profiling Metabolomics. *J. Postgenomics Drug Biomark. Dev.* **04**, (2014).
3. Evans, A. M., DeHaven, C. D., Barrett, T., Mitchell, M. & Milgram, E. Integrated, Nontargeted Ultrahigh Performance Liquid Chromatography/Electrospray Ionization Tandem Mass Spectrometry Platform for the Identification and Relative Quantification of the Small-Molecule Complement of Biological Systems. *Anal. Chem.* **81**, 6656–6667 (2009).
4. DeHaven, C. D., Evans, A. M., Dai, H. & Lawton, K. A. Organization of GC/MS and LC/MS metabolomics data into chemical libraries. *J. Cheminformatics* **2**, (2010).
5. Long, T. *et al.* Whole-genome sequencing identifies common-to-rare variants associated with human blood metabolites. *Nat. Genet.* **49**, 568–578 (2017).

Supplementary Figures

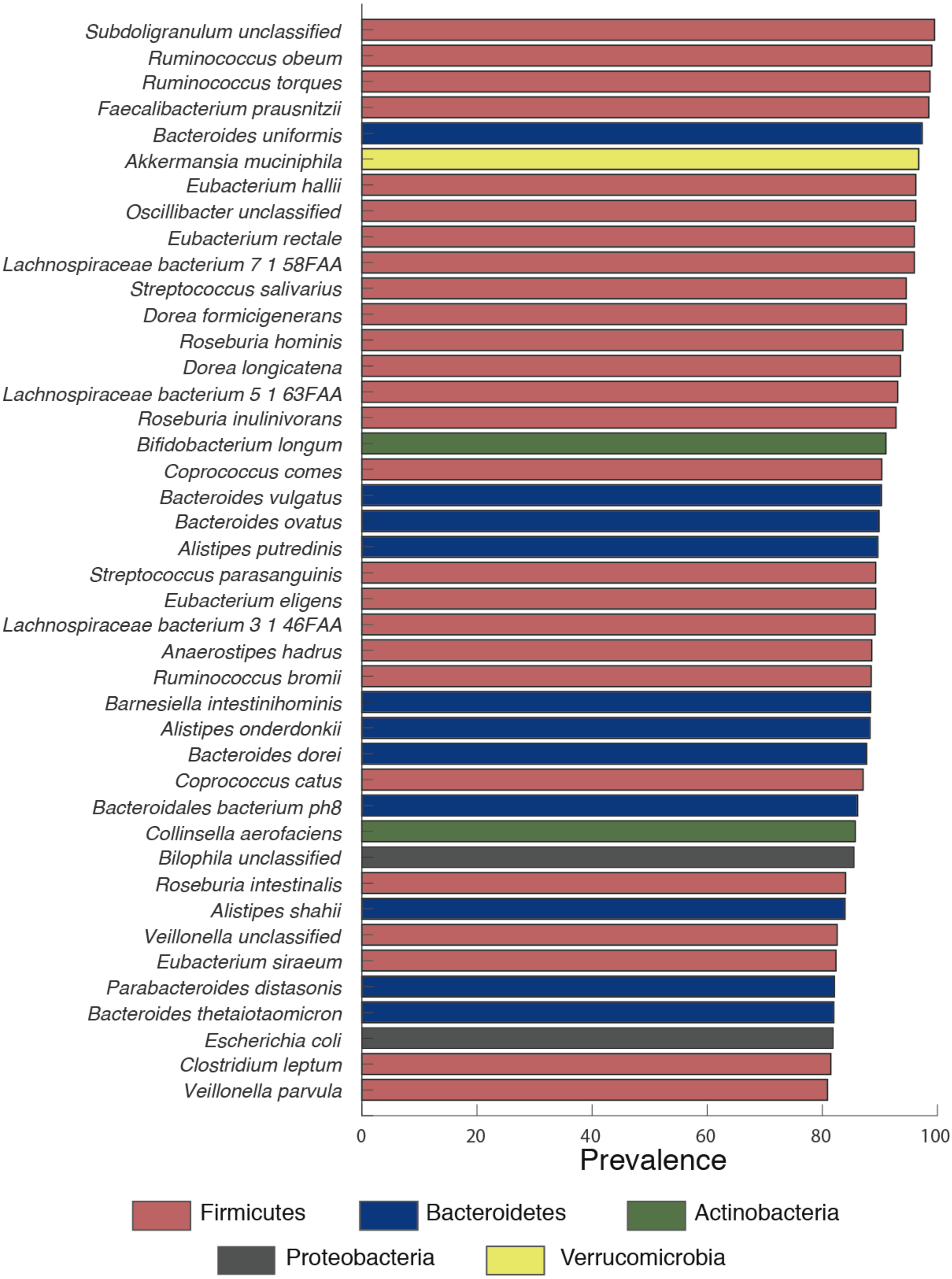

Supplementary Figure 1. Species detected in at least 80% of the population.

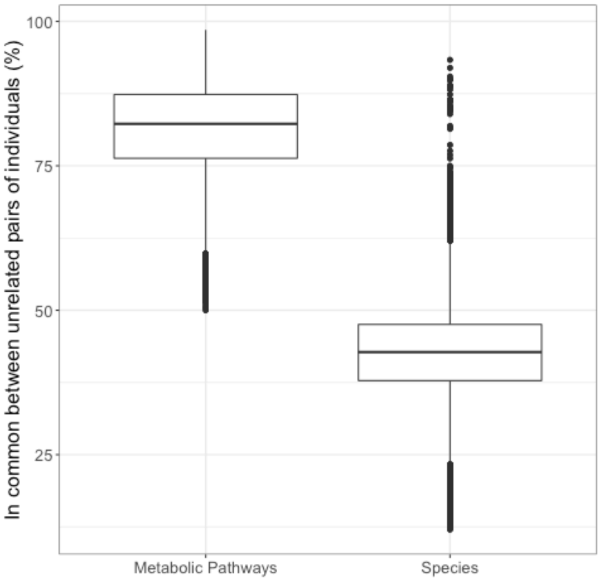

Supplementary Figure 2. Percentages of microbial metabolic pathways and species shared between pairs of unrelated individuals. The percentage of shared species/pathways was evaluated as the ratio between the number of species/pathways which were present in both members of the pair and the number of species/metabolic pathways which were present in at least one of the members of the pair. Pathways/species not measured in either individual were not considered.

The center line of the box plot indicates the median, limits of the box indicate the 25th and 75th percentile. The whiskers represent either 1.5 times the interquartile range (IQR) or the maximum/minimum data point if they are within 1.5 times the IQR. Points represents outliers outside 1.5 times the IQR.

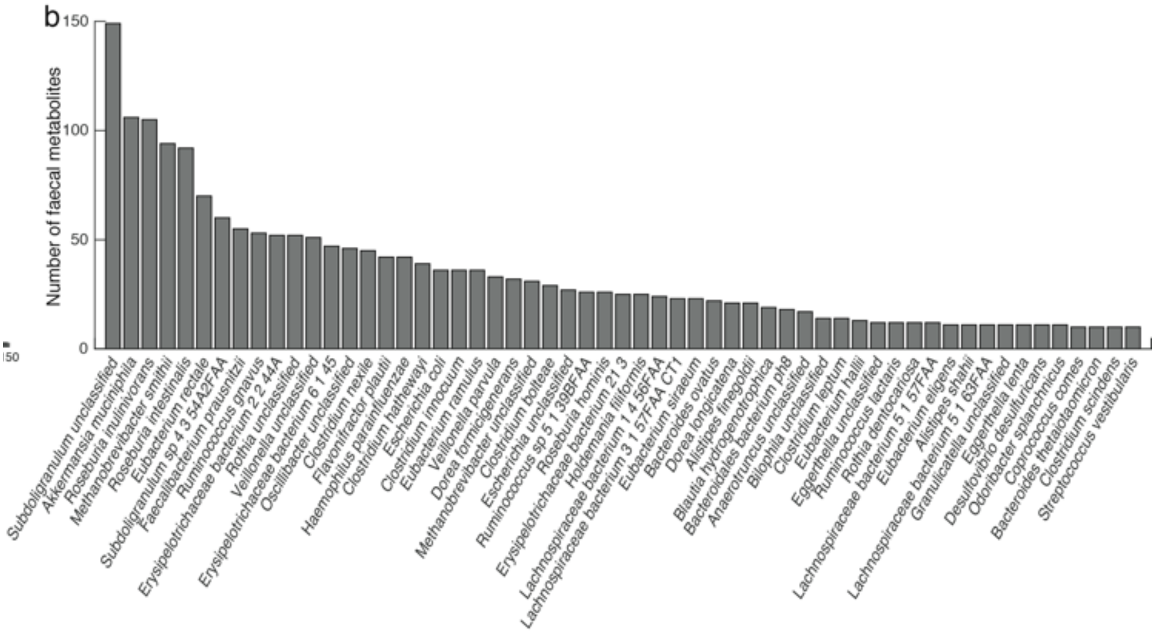

Supplementary Figure 3. Number of faecal metabolites significantly associated with microbial species.

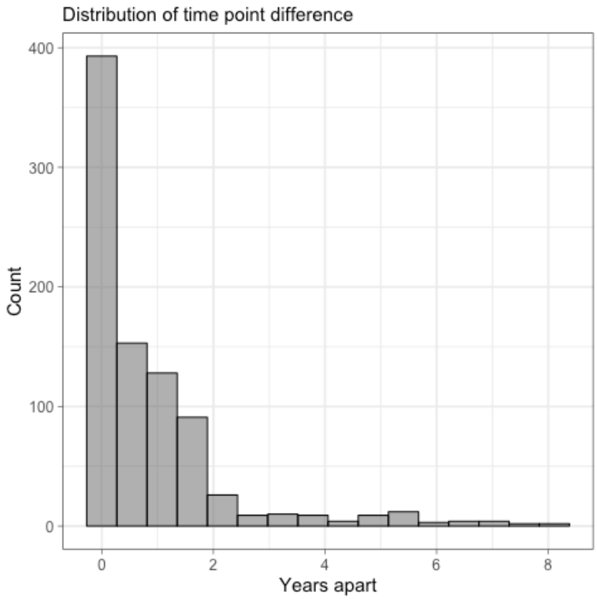

Supplementary Figure 4. Distribution of time point difference between metagenomic and blood metabolomic data. Mean difference was 0.9 years (median: 0.43, SD: 1.3).

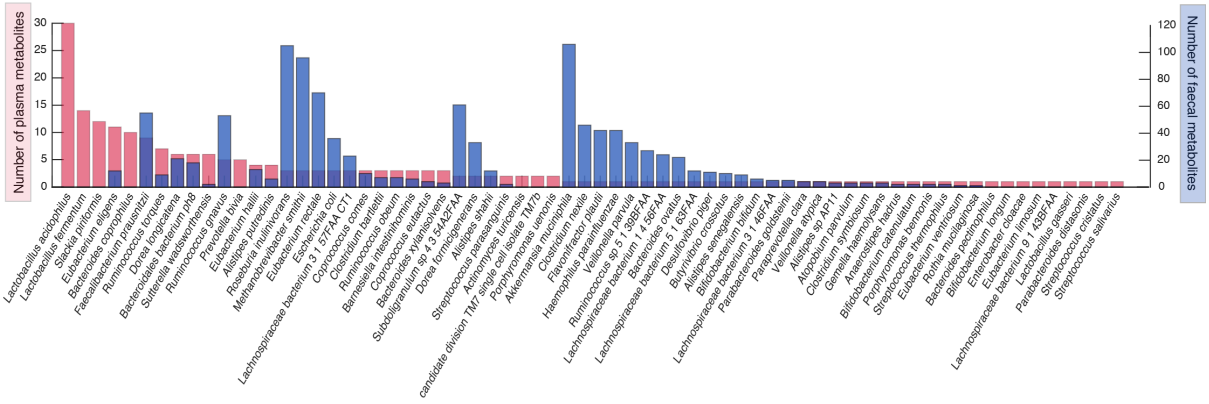

Supplementary Figure 5. Number of blood metabolites significantly associated with microbial species (red bars; left axis). The overlapping blue bars represent the number of faecal metabolites significantly associated with the same species (right axis).

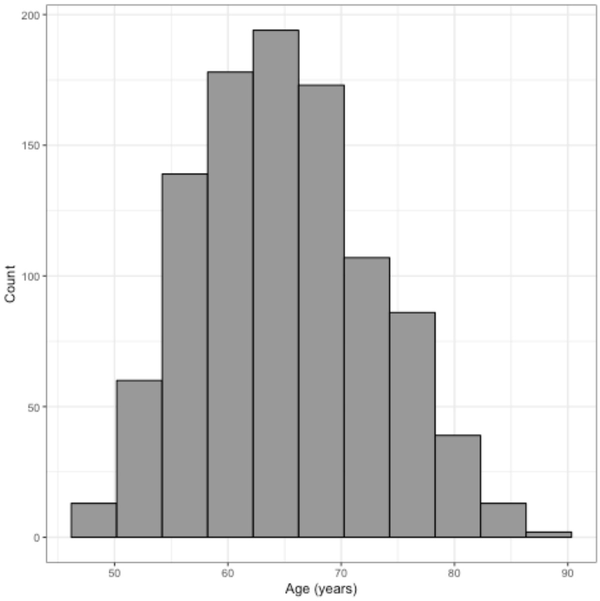

Supplementary Figure 6. Age distribution in the study dataset.

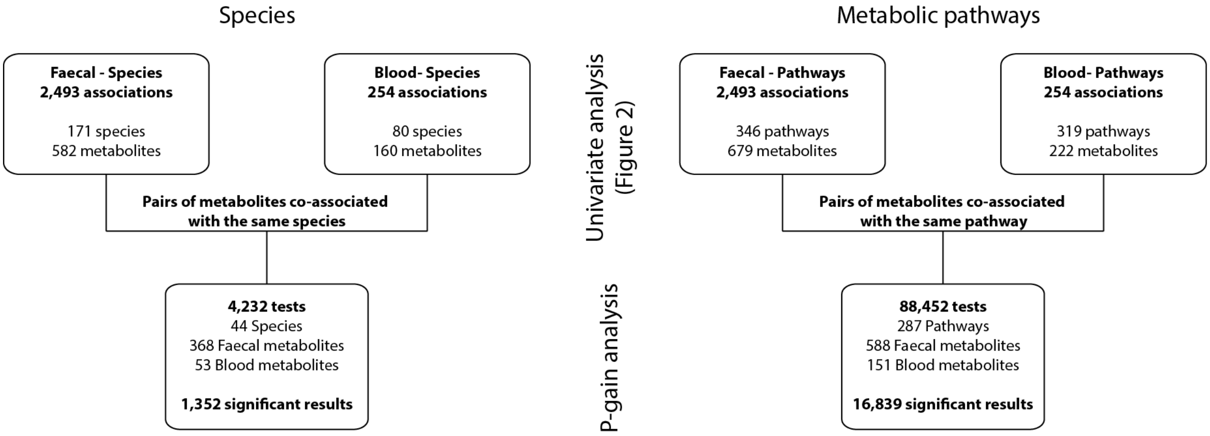

Supplementary Figure 7. Data selection process for the P-gain analysis.

Supplementary Tables

Supplementary Table 1. Population statistics for the individuals in the study dataset.

	Value
Age	65.0 ± 7.8 year
Sex (F/M)	965/39 (96.1/3.9 %)
MZ/DZ/Singletons	322/402/280
BMI	26.17 ± 4.71 kg m^{-2}
Total Fat (%)	40.00 ± 6.22
Visceral Fat (%)	38.99 ± 8.98

Supplementary Table 2. Intra-individuals' correlation of blood metabolomics profiles over time. The table reports, for measurements taken up to 10 years apart, the number of individuals used within each time frame (N), and the mean intra-individual correlations, along with their standard error (SD), and 1st-3rd interquartile (IQ) range.

Years apart	N	Mean	SD	1st-3rd IQ range
2	149	0.53	0.12	0.47-0.60
3	180	0.52	0.13	0.43-0.62
4	282	0.52	0.13	0.43-0.61
5	352	0.50	0.13	0.42-0.60
6	446	0.48	0.13	0.40-0.57
7	552	0.49	0.12	0.41-0.58
8	506	0.49	0.13	0.41-0.59
9	331	0.48	0.13	0.40-0.58
10	139	0.48	0.12	0.39-0.58

Supplementary Table 3. Number of associations observed when correcting for age at the sample collection, and number of associations that remain significant, at a 5% FDR, showing also concordant direction of effects, when age was not taken into account.

		Correcting for age	Overlap
Faeces	Species	2493	2237 (89.7%)
	Pathway	16133	15646 (97.0%)
Blood	Species	254	234 (92.1%)
	Pathways	2030	1980 (97.5%)

Supplementary Table 4. Number of associations observed, in a subset of 411 individuals, when not correcting for use of antibiotics, metformin and PPI at sample collection, and number of associations that remained significant (5% FDR), showing also concordant direction of effects, when the information on these three drugs was taken into account.

		**Without correction for drug intake**	**Overlap**
Faeces	Species	1000	917 (91.7%)
	Pathways	6292	5847 (92.9%)
Blood	Species	43	42 (97.7%)
	Pathways	569	498 (87.5%)

Supplementary Table 5. Number of species and microbial metabolic pathways which are associated to at least one metabolite in faeces/blood. "Shared" indicates the number of species and pathways, that are associated to at least one metabolite in faeces and in blood.

	**Faecal-specific**	**Blood-specific**	**Shared**
Microbial species	112	21	59
Microbial metabolic pathways	41	14	305

Supplementary Table 6. Results of the association study between threonate (in blood and faeces) and *Methanobrevibacter smithii* and three measures of adiposity. We used a linear mixed model to control for family structure. Age and sex were used as covariates.

**Predictor**	**Trait**	**N**	**Beta**	**SE**	**P**
threonate (Blood)	BMI	986	-0.48	0.12	3.2×10^{-5}
	Total Fat (%)	987	-0.41	0.10	4.3×10^{-5}
	Visceral Fat (%)	988	-0.48	0.11	2.6×10^{-5}
threonate (Faeces)	BMI	509	-0.12	0.09	0.181
	Total Fat (%)	515	-0.01	0.08	0.879
	Visceral Fat (%)	515	0.04	0.09	0.655
M. smithii	BMI	615	-0.07	0.04	0.074
	Total Fat (%)	614	-0.06	0.03	0.053
	Visceral Fat (%)	615	-0.09	0.04	0.013
