# Supplementary material for: Interplay between the human gut microbiome and host metabolism
Source: Nat Commun. 2019 Oct 3;10:4505. doi: 10.1038/s41467-019-12476-z (PMC6776654; doi:10.1038/s41467-019-12476-z)
Supplement: Supplementary file 3 — Description of Additional Supplementary Files [file 41467_2019_12476_MOESM3_ESM.docx]

**Description of Supplementary Files**

**File Name:** Supplementary Data 1

**Description:** Species contributing to the abundance of each microbial metabolic pathway.

**File Name:** Supplementary Data 2

**Description:** Results of the association study between microbial species and faecal metabolites.

**File Name:** Supplementary Data 3

**Description:** Results of the association study between microbial metabolic pathways and faecal metabolites.

**File Name:** Supplementary Data 4

**Description:** Results of the association study between microbial species and blood metabolites.

**File Name:** Supplementary Data 5

**Description:** Results of the association study between microbial metabolic pathways and blood metabolites.

**File Name:** Supplementary Data 6

**Description:** Results of the association study between microbial species and faecal metabolites (not age-adjusted).

**File Name:** Supplementary Data 7

**Description:** Results of the association study between microbial metabolic pathways and faecal metabolites (not age-adjusted).

**File Name:** Supplementary Data 8

**Description:** Results of the association study between microbial species and blood metabolites (not age-adjusted).

**File Name:** Supplementary Data 9

**Description:** Results of the association study between microbial metabolic pathways and blood metabolites (not age-adjusted).

**File Name:** Supplementary Data 10

**Description:** Results of the association study between microbial species and faecal metabolites (both adjusted and not-adjusted for drug intake).

**File Name:** Supplementary Data 11

**Description:** Results of the association study between microbial metabolic pathways and faecal metabolites (both adjusted and not-adjusted for drug intake).

**File Name:** Supplementary Data 12

**Description:** Results of the association study between microbial species and blood metabolites (both adjusted and not-adjusted for drug intake).

**File Name:** Supplementary Data 13

**Description:** Results of the association study between microbial metabolic pathways and blood metabolites (both adjusted and not-adjusted for drug intake).

**File Name:** Supplementary Data 14

**Description:** Association results with microbial metabolic pathways involving exactly the same named metabolite in both faeces and blood.

**File Name:** Supplementary Data 15

**Description:** Results for the P-gain statistic analysis (microbial species).

**File Name:** Supplementary Data 16

**Description:** Results for the P-gain statistic analysis (microbial metabolic pathways).

**File Name:** Supplementary Data 17

**Description:** Associations between adiposity measurements and faecal metabolites involved in a dialogue with blood threonate.

**File Name:** Supplementary Data 18

**Description:** Blood metabolites heritability from Long, T. et al.

**File Name:** Supplementary Data 19

**Description:** Metabolon platform used for faecal metabolites.

**File Name:** Supplementary Data 20

**Description:** Metabolon platform used for blood metabolites.
