# Supplementary material for: Interplay between the human gut microbiome and host metabolism
Source: Nat Commun. 2019 Oct 3;10:4505. doi: 10.1038/s41467-019-12476-z (PMC6776654; doi:10.1038/s41467-019-12476-z)
Supplement: Supplementary file 24 — Reporting Summary [file 41467_2019_12476_MOESM24_ESM.pdf]

natureresearch

Corresponding author(s): Mario Falchi (mario.falchi@kcl.ac.uk)

Last updated by author(s): Aug 21, 2019

Reporting Summary

Nature Research wishes to improve the reproducibility of the work that we publish. This form provides structure for consistency and transparency in reporting. For further information on Nature Research policies, see Authors & Referees and the Editorial Policy Checklist.

Statistics

For all statistical analyses, confirm that the following items are present in the figure legend, table legend, main text, or Methods section.

n/a	Confirmed	
☐	☒	The exact sample size (*n*) for each experimental group/condition, given as a discrete number and unit of measurement
☐	☒	A statement on whether measurements were taken from distinct samples or whether the same sample was measured repeatedly
☐	☒	The statistical test(s) used AND whether they are one- or two-sided<br>*Only common tests should be described solely by name; describe more complex techniques in the Methods section.*
☐	☒	A description of all covariates tested
☐	☒	A description of any assumptions or corrections, such as tests of normality and adjustment for multiple comparisons
☐	☒	A full description of the statistical parameters including central tendency (e.g. means) or other basic estimates (e.g. regression coefficient) AND variation (e.g. standard deviation) or associated estimates of uncertainty (e.g. confidence intervals)
☐	☒	For null hypothesis testing, the test statistic (e.g. *F*, *t*, *r*) with confidence intervals, effect sizes, degrees of freedom and *P* value noted<br>*Give P values as exact values whenever suitable.*
☒	☐	For Bayesian analysis, information on the choice of priors and Markov chain Monte Carlo settings
☒	☐	For hierarchical and complex designs, identification of the appropriate level for tests and full reporting of outcomes
☐	☒	Estimates of effect sizes (e.g. Cohen's *d*, Pearson's *r*), indicating how they were calculated

Our web collection on statistics for biologists contains articles on many of the points above.

Software and code

Policy information about availability of computer code

Data collection	No software was used
Data analysis	- YAMP [DOI: 10.1093/gigascience/giy072] was used pre-process the metagenomic data (Version 0.9.1, source code: https://github.com/alesssia/YAMP)<br>- PopPAnTe [DOI: 10.1186/s12864-017-3527-7] was used for the association study (Version 1.0.2, source code: https://github.com/alesssia/poppante)<br>- custom code to assess the dialogue between the gut and systemic host metabolism (source code: https://github.com/alesssia/microbiome_metabolome_interplay)

For manuscripts utilizing custom algorithms or software that are central to the research but not yet described in published literature, software must be made available to editors/reviewers. We strongly encourage code deposition in a community repository (e.g. GitHub). See the Nature Research guidelines for submitting code & software for further information.

Data

Policy information about availability of data

All manuscripts must include a data availability statement. This statement should provide the following information, where applicable:

- Accession codes, unique identifiers, or web links for publicly available datasets
- A list of figures that have associated raw data
- A description of any restrictions on data availability

Data generated during the study are available as Supplementary Data, while a web interface for querying the associations between microbiome and metabolites is available at: http://www.metabgut.org. Results are shown as interactive tables and can also be visualised graphically. Data on TwinsUK twin participants are available to bona fide researchers under managed access due to governance and ethical constraints. Raw data should be requested via our website (http://twinsuk.ac.uk/resources-for-researchers/access-our-data/) and requests are reviewed by the TwinsUK Resource Executive Committee (TREC) regularly. The raw

metagenomic sequences are available from the European Nucleotide Archive website (study accession number: PRJEB32731).

Field-specific reporting

Please select the one below that is the best fit for your research. If you are not sure, read the appropriate sections before making your selection.

☒ Life sciences ☐ Behavioural & social sciences ☐ Ecological, evolutionary & environmental sciences

For a reference copy of the document with all sections, see nature.com/documents/nr-reporting-summary-flat.pdf

Life sciences study design

All studies must disclose on these points even when the disclosure is negative.

Sample size	Sample study was not statistically predetermined. Metagenomics sequencing was performed on 1,054 randomly selected samples, while faecal and blood metabolomics was assessed in 479 and 859 individuals with metagenomics data, respectively.
Data exclusions	For metagenomic data, a principal component analysis evaluated using the taxonomic profiling was used to identify and discard ecologically abnormal samples (N=37). If sample scores were greater than 3 times the standard deviation on one of the first 10 principal components the sample was labelled as outlier and discarded. Finally, we removed individuals not of European ancestry (N=9, self-reported via questionnaire) resulting in 1,004 samples.
Replication	No replication study was carried on
Randomization	Not relevant for this study
Blinding	Not relevant for this study

Reporting for specific materials, systems and methods

We require information from authors about some types of materials, experimental systems and methods used in many studies. Here, indicate whether each material, system or method listed is relevant to your study. If you are not sure if a list item applies to your research, read the appropriate section before selecting a response.

Materials & experimental systems

n/a	Involved in the study	
☒	☐	Antibodies
☒	☐	Eukaryotic cell lines
☒	☐	Palaeontology
☒	☐	Animals and other organisms
☐	☒	Human research participants
☒	☐	Clinical data

Methods

n/a	Involved in the study	
☒	☐	ChIP-seq
☒	☐	Flow cytometry
☒	☐	MRI-based neuroimaging

Human research participants

Policy information about studies involving human research participants

Population characteristics	The study cohort included individuals of European ancestry and composed predominantly of middle-aged woman (96%, average age 65 years old).
Recruitment	The TwinsUK adult twin registry includes about 14,000 subjects, predominantly females, with disease and lifestyle characteristic similar to the general UK population [PMID: 11780939].
Ethics oversight	St. Thomas' Hospital Research Ethics Committee approved the study, and all twins provided informed written consent.

Note that full information on the approval of the study protocol must also be provided in the manuscript.
